# Disruption of a putative mitochondrial oxaloacetate shuttle protein in *Aspergillus carbonarius* results in secretion of malic acid at the expense of citric acid production

**DOI:** 10.1186/s12896-019-0572-0

**Published:** 2019-11-04

**Authors:** Lei Yang, Tore Linde, Abeer H. Hossain, Mette Lübeck, Peter J. Punt, Peter S. Lübeck

**Affiliations:** 1 Department of Chemistry and Bioscience, Section for Sustainable Biotechnology, Aalborg University, A.C. Meyers Vaenge 15, DK-2450 Copenhagen, SV Denmark; 2 Present address: AGC Biologics, Vandtaarnsvej 83B, DK-2860, Soeborg, Copenhagen, Denmark; 3 Dutch DNA Biotech BV, Padualaan 8, 3584CH, Utrecht, The Netherlands

**Keywords:** *Aspergillus carbonarius*, Mitochondrial transport protein, Citric acid, Malic acid, Metabolic engineering

## Abstract

**Background:**

In filamentous fungi, transport of organic acids across the mitochondrial membrane is facilitated by active transport via shuttle proteins. These transporters may transfer different organic acids across the membrane while taking others the opposite direction. In *Aspergillus niger*, accumulation of malate in the cytosol can trigger production of citric acid via the exchange of malate and citrate across the mitochondrial membrane. Several mitochondrial organic acid transporters were recently studied in *A. niger* showing their effects on organic acid production.

**Results:**

In this work, we studied another citric acid producing fungus, *Aspergillus carbonarius*, and identified by genome-mining a putative mitochondrial transporter MtpA, which was not previously studied, that might be involved in production of citric acid. This gene named *mtpA* encoding a putative oxaloacetate transport protein was expressed constitutively in *A. carbonarius* based on transcription analysis. To study its role in organic acid production, we disrupted the gene and analyzed its effects on production of citric acid and other organic acids, such as malic acid. In total, 6 transformants with gene *mtpA* disrupted were obtained and they showed secretion of malic acid at the expense of citric acid production.

**Conclusion:**

A putative oxaloacetate transporter gene which is potentially involved in organic acid production by *A. carbonarius* was identified and further investigated on its effects on production of citric acid and malic acid. The *mtpA* knockout strains obtained produced less citric acid and more malic acid than the wild type, in agreement with our original hypothesis. More extensive studies should be conducted in order to further reveal the mechanism of organic acid transport as mediated by the MtpA transporter.

## Background

Bio-based production of organic acids by microorganisms as e.g. filamentous fungi in a biorefinery has a high potential as a substitution of chemicals produced from crude oil [1]. The organic acids (e.g. malic acid and succinic acid) can easily be converted and used as building blocks for deriving different commodity and specialty chemicals and in the past decades, filamentous fungi as *Aspergillus niger* and *Aspergillus oryzae* have been used in industrial production of organic acids. Recently, *Aspergillus carbonarius* was reported to be an efficient organic acid producer and as such may have a potential for bio-based production of C4-dicarboxylic acids. When several genetic modifications were made to improve carbon flux towards dicarboxylic acid production (fumaric, succinic and malic acid), an increased production of citric acid was often observed [2–4]. This phenomenon might be a result of the transport of organic acids across the mitochondrial membrane where e.g. malic acid is transported to the mitochondria in exchange with citric acid. Recently, we identified a plasma membrane C_4_-dicarboxylate transporter, which was highly involved in the dicarboxylic acid production [5]. Overexpression of the transporter led to a significant increase of C_4_-dicarboxylic acid production and decreased citric acid production.

In the well-known organic acid producer, *A. niger*, which is phylogenetically related to *A. carbonarius*, the roles of mitochondrial transport of organic acid have been investigated in more details. *A. niger* is able to produce high amounts of organic acids (e.g. citric acid) from a broad range of substrates [6–8]. Considerable research efforts have been taken to reveal the mechanism of citric acid accumulation by the fungus and to redirect carbon flux from the production of citric acid towards other types of organic acids, e.g. itaconic acid production by genetically altered *A. niger* strains [9, 10]. In *A. niger,* citrate is produced in the mitochondria through the Tricarboxylic acid cycle (TCA cycle). Citrate is then transported out of the mitochondria into the cytosol by membrane bound citrate transporters [11]. A common belief is that these transporters are integral membrane proteins and function as antiporters where they exchange citrate from the mitochondria with other organic acids from the cytosol (e.g. cytosolic malate) [8, 12, 13]. It has been suggested that the onset of citric acid production is initiated in relation to the concentration of malate in the cytosol, which is then transported into the mitochondria in exchange for citrate. It was observed that malate was accumulated in the cytosol directly preceding start of production of citrate [12], and an *A. niger* strain transformed with malate dehydrogenase which, in theory, should produce elevated amounts of malate instead produced significantly higher amounts of citrate [8]. This supports the hypothesis that concentration of malate (or possible fumarate or succinate) in the cytosol will not result in secretion of these organic acids but instead produce an increased amount of citric acid due to a possible organic acid transport mechanism where C_4_-dicarboxylic acids are transported to the mitochondria in exchange with citric acid.

In *Saccharomyces cerevisiae,* it has been observed that the transport of citrate across the mitochondrial membrane is influenced not only by the concentration of malate, but also by the concentration of iso-citrate, succinate, and phosphoenolpyruvate [14, 15]. It is strongly suspected that the mitochondrial citrate transporter (CTP) is the primary transporter of citrate across the mitochondrial membrane in exchange of malate [16, 17]. In another *Aspergillus* species from the section Nigri, *Aspergillus luchuensis* the two putative CTP homologues *ctpA* and *ctpB* have been studied in some detail [18, 19]. The *ctpB* gene was shown to be non-expressed under citric acid production conditions, whereas *ctpA* was constitutively expressed in both *A. luchuensis* and *A. niger* [18, 20]. Although deletion of the *ctpA* gene resulted in serious growth effects of the resulting mutant strains, the effects on citric acid production were marginally [18, 19]. In addition to the mitochondrial citrate/malate antiporter, the role of two putative citrate/oxoglutarate antiporters was also studied in *A. luchuensis* [19] and one in *A. niger* [21]. In *S. cerevisiae* this antiporter is encoded by the *yhm2* gene responsible for exporting citrate from mitochondria to cytosol in exchange of oxoglutarate [22]. Also in this case one of the homologues, *yhmB*, was shown to be hardly expressed, while the closest homologue to *yhm2 (cocA)*, *yhmA* was also expressed constitutively in *A. luchuensis* and *A.niger,* [18, 20]. Deletion of *yhmB* showed no effect on citrate production, whereas *yhmA* and *cocA* deletion had a clear effect on citrate production, which was further aggravated in a conditional *ctpA*/*yhmA* double mutant, where malate and oxo-glutarate production was increased [19].

**Fig. 1:**
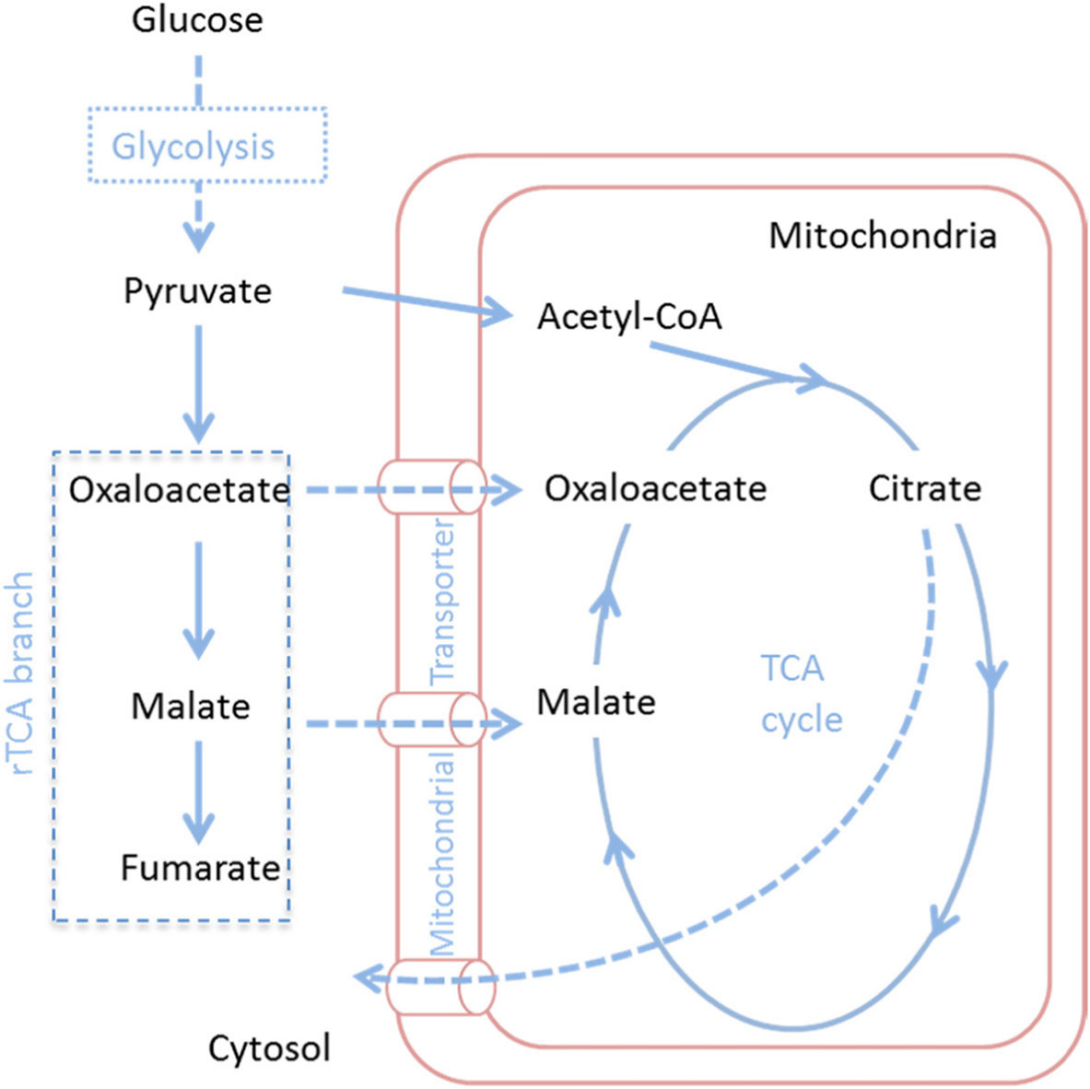
Metabolic pathway proposed for organic acid production by *A. carbonarius*

The hypothesis for the present study is based on the idea that when metabolic carbon-flux increases towards organic acids (e.g. dicarboxylic acids) other than citric acid in the cytosol in *A. carbonarius*, the accumulation of organic acids of interests will not happen due to transport of these acids (or their biosynthetic precursors) into the mitochondria by mitochondrial organic acid transporters in exchange of citrate (Fig. 1). If a transporter that is involved in exchange of organic acids is disrupted, the organic acid, which should be transported to the mitochondria in exchange with citrate, will remain in the cytosol, and in turn may be secreted to the exterior. The ability to reroute organic acid producing biocatalysts into production of more high-value acids would have great economic benefits since several organic acids are considered to be interesting building block chemicals [23]. In the present study, a putative mitochondrial oxaloacetate transporter gene was identified in *A. carbonarius* ITEM5010 based on a bioinformatic approach using characterized fungal organic acid transporters. Oxaloacetate is the key intermediate in the reductive tricarboxylic acid branch (rTCA branch) and the TCA cycle, which are highly involved in production of a number of organic acids e.g. citric acid, malic acid and succinic acid. In our work, the role of the oxaloacetate transport in organic acid production was investigated by disrupting the transporter gene and examining the changes in organic acid production by the knockout strain.

## Results

### Identification and expression of *mtpA* gene

Mitochondrial transporters of carboxylic acids are not well-characterized proteins in *Aspergillus* sp. To date only a few mitochondrial transporters of carboxylic acids have been studied in filamentous fungi [19, 24]. The number of candidate transporter proteins in a single fungal species is high, in yeast for example at least 34 different mitochondrial transporter proteins are known [25], and 39 are annotated in the *A. niger* genome [26]. The putative mitochondrial transporter gene investigated in the present study was identified using genome mining based on characterized mitochondrial proteins of fungal origin. For this purpose, homologues of the three genes identified in *S. cerevisiae* as being involved in citrate or citrate-intermediate transport, CTP1, YHM2 and OAC1 were identified by BLAST analysis in the genome of the most well characterized black *Aspergillus* genome, *A. niger* NRRL3 [27]. This resulted in the identification of 5 putative homologues, two for CTP1, two for YHM2 and one for OAC1. In Table 1, an overview of the expression data in *A. niger* available for these five genes in various publically available RNAseq data sets is given. From this analysis it is clear that the CTP1 and YHM2 homologues, *ctpA* and *yhmA*, are expressed to much higher and constitutive levels compared to *ctpB* or *yhmB,* respectively, confirming what was also already described by Kirimura et al. (2016) and Kadooka et al. (2018) [18, 19] . The OAC1 homologue was expressed to a similar level in all the studies represented in Table 1 (*A. niger* An14g06860 [20]. The putative *A. carbonarius* OAC1 orthologue, which we refer as *mtpA* was selected for further analysis. In order to confirm the expression of the *mtpA* gene in *A. carbonarius*, transcriptional analysis was carried out using reverse transcription polymerase chain reaction (RT-PCR). As shown in Additional file 1: Figure S1, a shorter fragment (~ 200 bp) was obtained by polymerase chain reaction (PCR) amplification of cDNA compared with genomic DNA, since the primers spanned the terminal part of the gene, where the cDNA in contrast to the genomic DNA contained no intron, confirming *mtpA* expression under these conditions.

**Table 1:** Transcription analysis of *A. niger* genes orthologous to the mitochondrial transporters identified in *A. carbonarius*

*A. carbonarius* ITEM 5010	Protein ID in *A. carbonarius* (JGI genome)	locus tag in *A. niger*	*A. niger* H915–1					*A. niger* AB 1.13	
			6 h_FPKM	12 h_FPKM	24 h_FPKM	36 h_FPKM	48 h_FPKM	75 h_RPKM	75 h_RPKM
			6 h	12 h	24 h	36 h	48 h	75 h	92 h
			citric acid titer at point of sampling (g/l)						
			≈ 3	≈ 18	≈ 50	≈ 86	≈ 118	2.5	7
*ctp1 (ctpA)*	139563	An11g11230	387.65	248.98	245.78	253.95	268.37	139,82	141,61
*ctp1 (ctpB)*	515063	An18g00070	3.70	0.83	3.24	0.54	1.18	0,54	0,56
*yhmA*	398055	An09g06670	605.39	406.06	315.49	532.38	395.27	386,26	329,29
*yhmB*	399724	An02g11090	36.37	55.64	9.04	41.48	76.31	0.07	0.06
*mtpA*	209833	An14g06860	70.50	35.61	44.76	38.02	38.64	58,62	59,27

*A. niger* H915–1- a citric acid producing strain [28]; *A. niger* AB1.13 – a commonly laboratory strain [20]. RPKM (Reads Per Kilobase of target per Million mapped reads) and FPKM (Fragments Per Kilobase of target per Million mapped reads) values were calculated according to the method presented by Mortazavi et al. [29], in order to normalize data for gene length

### Generation of knockout strains and southern blotting

**Fig. 2:**
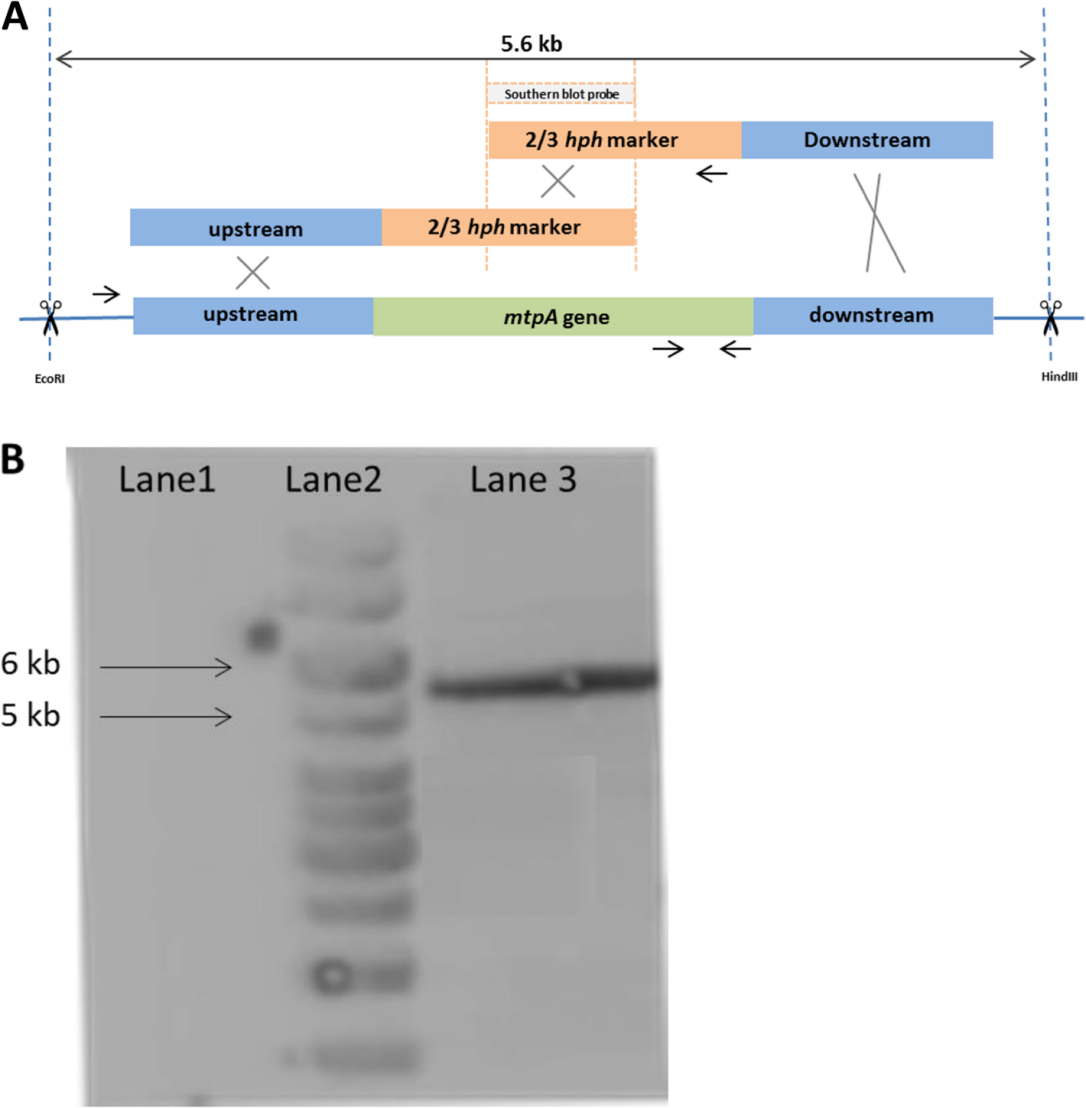
Verification of *mtpA* gene disruption in the transformant **a**) Disruption of *mtpA* gene with bipartite substrates in *A. carbonarius*. **b** Southern blotting analysis. Lane 1 hydridization of the probe to the genomic DNA fragment (from the wild-type) digested by *EcoRI* and *HindIII*, Lane 2, DNA ladder, Lane 3, hydridization of the probe to the genomic DNA fragment (from *mtpA-1* transformant) digested by *EcoRI* and *HindIII*

The putative organic acid transporter gene *mtpA* in *A. carbonarius* ITEM 5010 was knocked out using a bipartite approach to create knockout strains. Disruption of the target gene in the selected transformants was first verified by PCR (data not shown). This was done with a forward primer binding upstream to the gene in the genome paired with a reverse primer binding to the inserted hygromycin gene, so generation of the predicted PCR product indicated that the transporter gene was replaced by the hygromycin resistant gene (Fig. 2a). In total, 32 transformants were obtained, and of these, 6 transformants were verified as positive knockout giving a gene targeting efficiency of 20%, Southern blot hybridization was then performed on the selected transformant *mtpA-1*, and the hybridization of probe only occurred with the expected DNA fragment as shown in Fig. 2b, confirming successful gene disruption via homologous recombination without any ectopic integration in other sites of the genome.

### Organic acid production

**Fig. 3:**
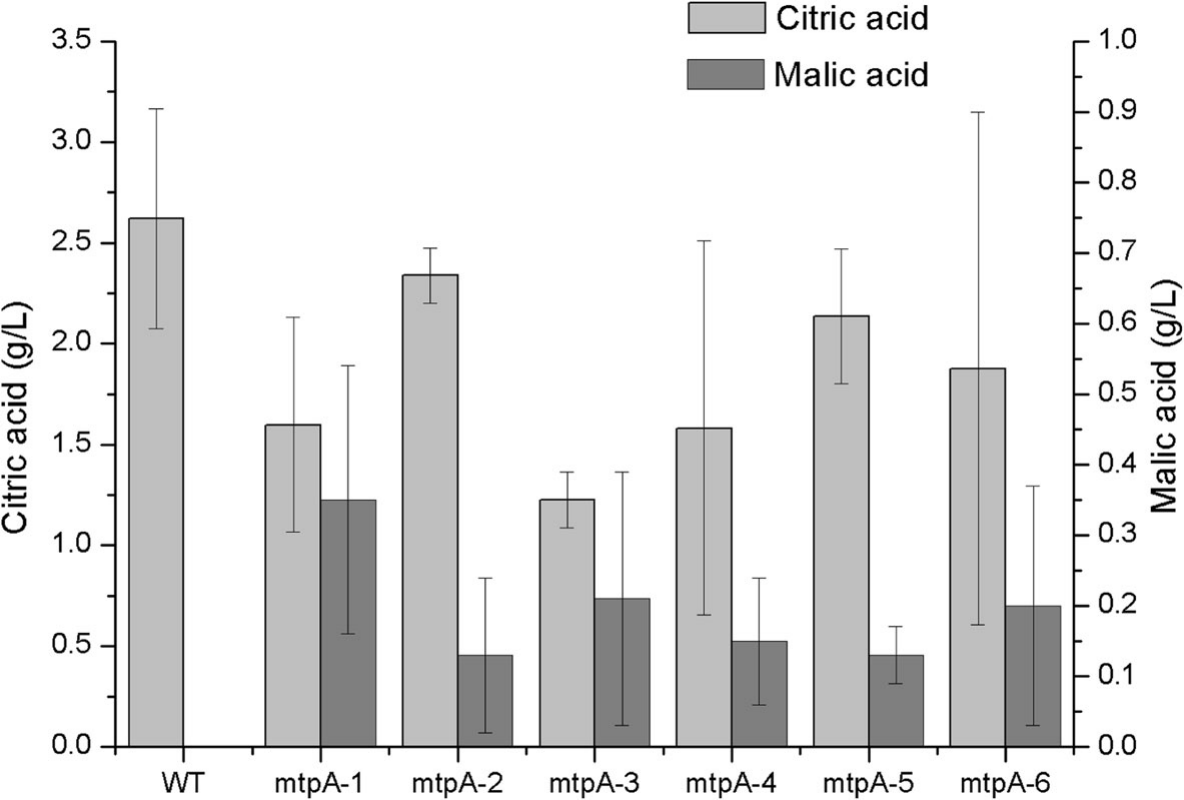
Organic acid production by *mtpA* transformants (Error bars shows standard error of the mean)

All 6 putative *mtpA* disruption strains were grown in shake flask cultures and analyzed for organic acid production by High-performance liquid chromatography (HPLC) analysis. As shown in Fig. 3, citric acid and malic acid were analyzed during the cultivation. All the transformants produced less citric acid than the wild type after day 5, meanwhile, a low but significant amount of malic acid was also detected from the culture with transformants but not from the wild type. No other major organic acid peaks were detected in the HPLC profile. The transformant *mtpA-1* that was verified via southern blotting was also analyzed in a pH controlled fermentation. As shown in Table 2, it consumed 29 g/l glucose and produced 1.6 g/l citric acid and 0.35 g/l malic acid while the wild type consumed 31 g/l glucose and produced 2.6 g/l citric acid and no malic acid.

**Table 2:** Effects of disrupting *mtpA* gene on organic acid production by *A. carbonarius* in shake flask fermentation

Strains/Titer (g/L)	Glucose consumption	Citric acid	Malic acid
WT	31.0 ± 1.27	2.6 ± 0.54	n.d.
*mtpA-1*	29.2 ± 7.83	1.6 ± 0.53	0.35 ± 0.19

(Note: Cultivations were performed in triplicates, showing standard error of the mean, n.d. – non-detectable level in HPLC)

## Discussion

A putative mitochondrial oxaloacetate transporter gene *mtpA* was identified in *A. carbonarius* and knocked out. The *mtpA* mutant was investigated for its impact on production of citric acid and malic acid. Disruption of the *mtpA* gene resulted in a reduction in citric acid production and secretion of malic acid in the culture medium. The hypothesis of this research study was that the disruption of this organic acid transporter could reduce the production of citric acid and simultaneously increase accumulation of malic acid or other dicarboxylic acids. Oxaloacetate is used in both cytosolic rTCA branch and mitochondrial TCA cycle respectively to produce malic acid and citric acid (Fig. 1). The deletion of *mtpA* may stop the transport of oxaloacetate into TCA cycle in mitochondria and save more carbon flux towards downstream rTCA branch in cytosol, which could be the reason for the measured increase in malic acid. However, only mild effects on production of citric acid and malic acid have been obtained. Apart from the fact that these effects may be larger in high citric acid production strains like *A. niger* H915–1 [28], a reason for this could also be that the MtpA membrane protein is part of a more complex network of transmembrane proteins involved in antiport and transport of different organic acids over the mitochondrial membrane. Similarly modest effects on organic acid production were also reported in the case of knocking out mitochondrial citrate transporters in *A. niger* and *A. luchuensis*. Citric acid production was also only slightly affected in *ctpA* knockout strain suggesting that other transporters also play important roles in the export of citric acid during the production phase [18]. On the other hand, deletion of the citrate/oxoglutarate antiporter *yhmA* had a more serious effect on citric acid and malic acid production, but also showed serious pleiotropic effects, making conclusion about its role in citric acid production somewhat ambiguous [18, 19]. It should also be noted that the expression of the genes encoding each of the citric acid related mitochondrial transporters is similar in high and low citric acid production strains and conditions (Table 2).

In the study presented here, the effect of deletion of the putative oxaloacetate carrier MtpA may be somewhat moderate due to a rerouting of the organic acid fluxes by other mitochondrial dicarboxylate transporters that can transport malate from the cytosol to mitochondria, where malate is the precursor for oxaloacetate synthesis in the TCA cycle and thus for citric acid production. Since shaking cultures, as performed in this study, are prone to variation, we carried out replicate cultures to determine the statistical relevance of the results obtained. A further more definitive conclusion would require further investigations, including controlled fermentation experiments in different scales and under different optimized conditions or knockouts of *mtpA* in combination with other putative transporter genes. Also more detailed in vitro transporter specificity studies as performed for *A. luchuensis* CtpA and YhmA [19], could be performed for MtpA to determine kinetic transport parameters and substrate specificity. In these studies, the function of a transporter was defined in the presence or absence of substrates or even radioactive labeled substrates as was recently performed for the first time for a fungal mitochondrial transporter [30].

## Conclusions

A putative oxaloacetate transporter gene was identified in *A. carbonarius* and further investigated on its effects on citric and malic acid production. The *mtpA* knockout mutant obtained secreted malic acid at the expense of citric acid which was the only secreted organic acid in the wild type, in agreement with our original hypothesis for the effect of gene disruption on organic acid production.

## Methods

### Strains and growth conditions

*A. carbonarius* ITEM 5010 was used as the parental strain to construct transformants. All the fungal strains were cultivated as previously described in [31] . *Escherichia coli* strain DH5α was used as host for plasmid propagation.

### Genomic DNA extraction and plasmid construction

Genomic DNA extraction from freshly grown mycelia and measurement of DNA concentration were carried out as described in [31]. The knockout plasmid pSB4.1.1 containing *RP27-hph-βT* for hygromycin resistance, origin of replication and ampicillin resistance for growth in *E.coli* and a specific cassette facilitating simpleUSER cloning was constructed as described in [2]. All primers used in the study were designed with the primer software Primer3 [32] (Table 3). In the knock out plasmid pSB4.1.1-Antiporter upstream and downstream regions to the target gene were identified in the *A. carbonarius* genome provided by JGI. The upstream and downstream flanking regions with the size of 1 kb were selected for efficient homologous recombination [33] and amplified with primers containing uracil tails (primers 1–4, Table 3). Genomic DNA from *A. carbonarius* was used as template. The flanking regions of gene were amplified by PCR using Pfu turbo cx polymerase (Agilent) and 65 °C as annealing temperature. The obtained PCR products were then ligated to the simpleUSER cassette in pSB4.1.1 that was already digested with the restriction enzyme *PacI* and the nicking enzyme *Nb.BbvCI* to create the complementary overhangs as previously described [2]. Through self-assembly the PCR fragments were cloned into the plasmid followed by transformation of *E. coli* with the plasmid for further propagation. Plasmid extraction with the GeneJET plasmid miniprep kit (ThermoFisher Scientific) was carried out according to the manufacturer’s protocol. All plasmids were verified by Sanger sequencing using the sequencing service from StarSEQ (Mainz, Germany).

**Table 3:** Primers used in the study

Name	No.	Sequence (5′ → 3′)	Annotation
*mtpA* up-fw-U	1	GGGTTTAAUAGACATACCGTCGACCTTGG	Amplify upstream region of *mtpA*
*mtpA* up-rv-U	2	GGACTTAAUGAGGGTGAGTCTGGCAGAAG	Amplify upstream region of *mtpA*
*mtpA* do-fw-U	3	GGCATTAAUTCAGTTTTGCATGGTTGAGC	Amplify downstream region of *mtpA*
*mtpA* do-rv-U	4	GGTCTTAAUGCGGGTGGTATTCTCTGTGT	Amplify downstream region of *mtpA*
Bipart-1 rv	5	GATGTTGGCGACCTCGTATT	Amplify 1st bipartite fragment
Bipart-2 fw	6	GATGTAGGAGGGCGTGGATA	Amplify 2nd bipartite fragment
*mtpA* up-fw	7	AGACATACCGTCGACCTTGG	Amplify 1st bipartite fragment
*mtpA* do-rv	8	GCGGGTGGTATTCTCTGTGT	Amplify 2nd bipartite fragment
*mtpA* ko-ch-fw	9	GTCGCAAGCTTCAACTTTCC	Check for positive knockout of *mtpA*
*mtpA* ko-ch-rv	10	TATTGGAGAGCAAGGGATGG	Check for positive knockout of *mtpA*
Hph-Fw	11	GATGTAGGAGGGCGTGGATA	Amplify *hph* marker
Hph-Rv	12	GATGTTGGCGACCTCGTATT	Amplify *hph* marker
*mtpA*-fw	13	CAAGTTCTCCTTGAGTGAGTCG	Transcription analysis of *mtpA*
*mtpA*-rv	14	TCGACTGCCTTTACAAGACC	Transcription analysis of *mtpA*

### Protoplast transformation

Protoplasts were prepared from wild type *A. carbonarius* following the procedure described in [4]. Bipartite PCR fragments were made from the knockout plasmids pSB4.1.1-Antiporter and primer 5–8 (Table 3) and prepared for transformation as described in [31]. Transformation was done in 100 μl aliquots of protoplasts according to the method described by [4]. Transformants carrying hygromycin resistance were isolated and transferred to PDA plates containing hygromycin. All the transformants were preserved by mixing spores suspension with 20% glycerol (final concentration in cryostock) and stored at − 80 °C. Validation of positive knockouts was carried out by PCR using primers 9–10 (Table 3).

### Transcriptional analysis of *mtpA* gene

Transcription of the *mtpA* gene was analyzed by reverse-transcription PCR (RT-PCR). Total RNA purification and cDNA synthesis were prepared as previously described [5]. The cDNA was then used as template in PCR with *mtpA* gene specific primers no. 13–14 for transcription analysis.

### Southern blotting of *mtpA* transformant

Southern blotting was carried out on a single *mtpA* transformant for confirmation of *mtpA* gene disruption. As shown in Fig. 2a, the hybridization probe was prepared by amplifying a part of the *hph* marker gene with primers 11–12 (Table 3) and labeled using a North2South chemiluminescent detection kit (Thermo Fisher Scientific). Genomic DNA of the transformant was digested by *EcoRI* and *HindIII* and hybridized with the probe. Southern blotting was then performed using Whatman Turboblotter transfer system (GE healthcare life sciences) and detected by using a Pierce Chemiluminescent Nucleic acid detection module Kit (Thermo scientific). The blot was imaged in Thermo ECL imager.

### Fermentation and shake flask cultivation condition

Fermentation was performed in 50 ml Erlenmeyer flasks in 10 ml media in triplicates. Fresh spore-suspension was added to 10 ml pre-culture medium (Yeast extract 3.6 g/l and peptone 10 g/l) to a final concentration of 2 × 10^5^ spores/ml. Pre-culture was incubated at 25 °C, 200 rpm and 2.5 cm amplitude for 48 h (KS 4000 I control, IKA). For the pH controlled fermentation, the mycelia pellets from the pre-culture were transferred to Erlenmeyer flasks with cotton stoppers and 10 ml production media [34] containing 100 g/l glucose, 2 g/l (NH_4_)_2_SO_4_, 0.15 g/l KH_2_PO_4_, 0.15 g/l K_2_HPO_4_, 0.1 g/l MgSO_4_∙7H_2_O, 0.1 g/l CaCl_2_∙2H_2_O, 0.005 g/l NaCl, 0.1 g/l ZnSO_4_, 0.005 g/l FeSO_4_·7H_2_O and 30 g/l CaCO_3_(for pH maintenance). Flasks were incubated at 25 °C, 200 rpm. The supernatant obtained from each fermentation culture was prepared as described by [35]. 1 ml sample from each flask was taken at day 5. 50 μl 50% H_2_SO_4_ was added and the mixture was heated to 80 °C and incubated for 15 min. The samples were then cooled down to the room temperature followed by centrifugation at 8000 rpm for 1 min. The supernatant was filtered through 0.45 μM HPLC-grade regenerated cellulose membrane filters for HPLC analysis. 250 μl of the filtrate were analyzed for the content of sugars and organic acids by HPLC (Dionex Ultimate 3000-LC system) with the conditions described in [31].

### Statistical analysis

Comparison of results from triplicates were analyzed by *t*-test with a significance level of *p* < 0.05. Error bars on figures are standard error of the mean.

## Additional file

**Additional file 1: Figure S1.** Transcriptional analysis of the mtpA gene.

## Data Availability

All the data and material presented in the article are available from the corresponding author upon reasonable request.

## Abbreviations

CTP: Citrate transporter
HPLC: High-performance liquid chromatography
PCR: Polymerase chain reaction
rTCA branch: Reductive tricarboxylic acid branch
RT-PCR: Reverse transcription polymerase chain reaction
TCA cycle: Tricarboxylic acid cycle
