# Supplementary material for: Disruption of a putative mitochondrial oxaloacetate shuttle protein in Aspergillus carbonarius results in secretion of malic acid at the expense of citric acid production
Source: BMC Biotechnol. 2019 Nov 4;19:72. doi: 10.1186/s12896-019-0572-0 (PMC6829807; doi:10.1186/s12896-019-0572-0)

**Disruption of a putative mitochondrial oxaloacetate shuttle protein in *Aspergillus carbonarius* results in secretion of malic acid at the expense of citric acid production**

Lei Yang^1¶^, Tore Linde^1,2¶^, Abeer H. Hossain^3^, Mette Lübeck^1^, Peter J. Punt^3^, Peter S. Lübeck^1^§

1) Department of Chemistry and Bioscience, Section for Sustainable Biotechnology, Aalborg University, A.C. Meyers Vaenge 15, Copenhagen SV, DK-2450, Denmark

2) Present address: AGC Biologics, Vandtaarnsvej 83B, DK-2860, Soeborg, Copenhagen Denmark

3) Dutch DNA Biotech BV, Padualaan 8, 3584CH Utrecht, The Netherlands

^¶^Tore Linde and Lei Yang contributed equally to this work

§Corresponding author – Email: psl@bio.aau.dk – Telephone: +45 99402590

**Fig. S1** Transcriptional analysis of *mtpA* gene Lane 1, partial *mtpA* gene amplified from cDNA template; Lane 2, 100 bp DNA ladder; Lane 3 partial *mtpA* gene amplified from genomic DNA template (containing one intron)


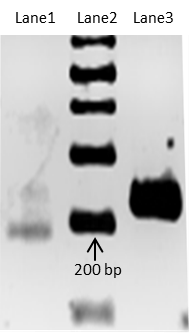

Supplement: Supplementary file 1 — Additional file 1: Figure S1. Transcriptional analysis of the mtpA gene. [file 12896_2019_572_MOESM1_ESM.docx]
